# Steroid-sparing strategies in polymyalgia rheumatica: a systematic review and meta-analysis of tocilizumab with practical guidance for tapering

**DOI:** 10.1186/s41927-026-00625-z

**Published:** 2026-04-25

**Authors:** Musa Khan Bungish, Maheen Mohsan, Hareem Farooq, Muhammad Talha Shaukat, Wania ur Rehman, Ahmed Talal Murtaza, Arooj Fatima, M. Hasnat Akhtar, Hassan Farooq, Amna Mufti, Salman Sarwar, Aqeeb Ur Rehman, Aleenah Mohsin

**Affiliations:** 1 Department of Internal Medicine, CMH Kharian Medical College, Kharian Cantt, Kharian, Pakistan; 2 College of Medicine, Shifa Tameer-e-Millat University, Islamabad, Pakistan; 3 Department of Internal Medicine, University of Alabama at Birmingham, Birmingham, USA; 4 Department of Internal Medicine, King Edward Medical University, Lahore, Pakistan; 5 Department of Internal Medicine, Ameer-ud-Din Medical College, Lahore, Pakistan; 6 Department of Internal Medicine, Allama Iqbal Medical College, Lahore, Pakistan; 7 Department of Internal Medicine, Services Institute of Medical Sciences, Lahore, Pakistan

**Keywords:** Polymyalgia rheumatica, Tocilizumab, Interleukin−6 inhibitors, Glucocorticoids, Systematic review, Meta-analysis

## Abstract

Polymyalgia rheumatica (PMR) is an inflammatory rheumatic condition primarily managed with glucocorticoids (GCs), but prolonged GC use carries significant adverse effects. Tocilizumab, an interleukin−6 receptor inhibitor, has emerged as a promising steroid-sparing therapy for patients with relapsing or GC-dependent PMR. We conducted a systematic review and meta-analysis according to PRISMA guidelines to assess the efficacy and safety of tocilizumab in PMR. Four randomized controlled trials (RCTs) involving 317 patients were included in the meta-analysis, and one multicentre observational cohort was narratively synthesized to provide supportive real-world evidence. Relapse outcomes were categorized as short-term (≤ 12 weeks), medium-term (13–26 weeks), and long-term (> 26 weeks). Pooled analysis showed that tocilizumab significantly reduced short-term relapse rates (OR: 0.22, 95% CI: 0.12–0.41; I² = 22.5%) and medium and long-term relapse rates (OR: 0.19, 95% CI: 0.10–0.35; I² = 27.1%) compared to placebo. Tocilizumab also significantly lowered cumulative GC dose (SMD: -0.78, 95% CI: -1.21 to -0.23; I² = 84.5%). Severe relapse showed a non-significant trend favoring tocilizumab, and safety outcomes were comparable between groups (OR: 0.57, 95% CI:0.27–1.18; I² = 30.0%). The included observational study supported these results, demonstrating high rates of successful tapering (77% to ≤ 5 mg at six months; 97% at twelve months) with few adverse events. This updated synthesis supports tocilizumab as an effective steroid-sparing option in PMR and provides practical tapering guidance to assist clinicians in managing relapsing or GC-dependent cases.

**Supplementary Information:**

The online version contains supplementary material available at 10.1186/s41927-026-00625-z.

## Introduction

Polymyalgia rheumatica (PMR) is a chronic inflammatory rheumatic disorder characterized by bilateral shoulder and hip girdle pain, prolonged morning stiffness, and restricted range of motion. It primarily affects individuals over 50 years of age, with a higher prevalence among women and people of Northern European ancestry [1–3]. Elevated inflammatory markers, including erythrocyte sedimentation rate (ESR) and C-reactive protein (CRP), are common laboratory findings [1].

Although the precise pathogenesis remains unclear, increasing evidence points to immune-mediated pathways and aberrant cytokine signaling, particularly elevated interleukin-6 (IL-6) levels, as key drivers of inflammation [4–6]. Genetic predisposition and environmental factors also contribute to disease onset and regional variability [7–9].

Glucocorticoid (GC) therapy, usually with oral prednisone, remains the cornerstone of PMR treatment [10–12]. While highly effective for controlling inflammation and symptoms, long-term GC use is associated with significant adverse effects, including osteoporosis, diabetes, hypertension, cataracts, and increased infection risk [12]. Agents such as Vitamin D have been used with mixed results [13]. Methotrexate has been investigated as a steroid-sparing agent with modest benefit [14, 15], whereas non-steroidal anti-inflammatory drugs (NSAIDs) are generally ineffective for sustained remission [9]. Consequently, alternative strategies to reduce GC exposure while maintaining disease control are a major unmet need in PMR management.

Tocilizumab, a humanized monoclonal antibody targeting the IL-6 receptor, has emerged as a promising biologic option for patients with relapsing PMR or those who are intolerant of prolonged steroid therapy [16]. By inhibiting IL-6 signaling, tocilizumab reduces downstream inflammatory cytokine production and modulates T and B cell activity [16]. A related IL-6 receptor inhibitor, sarilumab, was recently evaluated in the SAPHYR trial, which demonstrated significant steroid-sparing efficacy and higher rates of sustained remission in patients with relapsing PMR [17]. While sarilumab and tocilizumab share the same IL-6 receptor target, their evidence bases differ, sarilumab has been studied exclusively in PMR, whereas tocilizumab evidence remains distributed across PMR-specific trials and PMR subgroups within broader inflammatory cohorts. Including SAPHYR in this context strengthens the mechanistic rationale for IL-6 receptor blockade in PMR and highlights the need to consolidate PMR-focused outcomes for tocilizumab [17]. Although not currently included in routine PMR treatment guidelines, its efficacy has been demonstrated in trials such as the GiACTA study, which enrolled patients with giant cell arteritis (GCA) with concomitant PMR features [18, 19].

Recently, two systematic reviews and meta-analyses published in early 2025 synthesized the existing randomized controlled trial (RCT) evidence for tocilizumab in PMR and confirmed its steroid-sparing benefits [20, 21]. Another review was published in letter format, however it contained a formal systematic synthesis of RCT data relevant to PMR [22]. All of these reviews were limited to controlled trials and did not incorporate relevant high quality, real world data available at the time, specifically, the multicenter observational cohort by Assaraf et al. (2024), which reported outcomes in 53 patients with glucocorticoid-dependent PMR treated with tocilizumab in routine clinical practice [23]. Incorporating this cohort provides valuable context for tapering feasibility and safety outside trial conditions.

Therefore, we conducted an updated systematic review and meta-analysis to consolidate the most recent RCT evidence and integrate supporting real-world data to provide a more comprehensive, practice-oriented assessment of tocilizumab’s efficacy, steroid-sparing potential, and safety in PMR. This review aims to address gaps left by prior analyses and offer clinicians clearer guidance for managing patients with relapsing or GC-dependent PMR, including a proposed evidence-based tapering framework derived from the pooled results and supportive real-world outcomes.

## Methods

This systematic review and meta-analysis was conducted according to the *Cochrane Handbook for Systematic Reviews of Interventions* [24] and reported in line with the *Preferred Reporting Items for Systematic Reviews and Meta-Analyses* (PRISMA) statement [25]. The protocol was prospectively registered in PROSPERO (CRD42024629228).

### Data sources and search strategy

We systematically searched MEDLINE, Embase, the Cochrane Central Register of Controlled Trials (CENTRAL), and ClinicalTrials.gov from inception through June 2025. Medical Subject Headings (MeSH) and free-text terms included “tocilizumab” and “polymyalgia rheumatica”. Additionally, we manually screened the reference lists of included studies and relevant systematic reviews to identify any further eligible studies, including relevant real world observational data. Abstracts from major rheumatology congresses (ACR and EULAR) were also screened. Relevant abstracts were incorporated narratively when they reported PMR specific outcomes but lacked sufficient quantitative detail for meta-analysis.

### Eligibility criteria

Eligible studies included randomized controlled trials (RCTs) comparing tocilizumab plus corticosteroids with placebo plus corticosteroids in patients diagnosed with polymyalgia rheumatica (PMR). In addition, high-quality observational cohort studies reporting clinical outcomes of tocilizumab in glucocorticoid dependent PMR were included narratively to supplement the RCT evidence. Studies involving animals, quasi-randomized designs, or reports lacking extractable PMR-specific outcomes were excluded. No language restrictions were applied.

Because some tocilizumab trials enrolled mixed PMR and GCA populations, these studies were included when PMR manifestations were a predefined component of the disease spectrum and PMR relevant clinical outcomes were reported, even if not available as fully separable PMR-only subgroups. No GCA-only outcomes were extracted or pooled. This approach aligns with prior PMR focused systematic reviews, which similarly incorporated trials with PMR features arising within GCA cohorts while restricting analyses to PMR relevant endpoints. This methodology minimizes heterogeneity related to the higher glucocorticoid requirements typical of GCA while acknowledging the clinical overlap between PMR and GCA.

Non-original articles (e.g., reviews, editorials) were excluded from the evidence synthesis but could be cited for contextual background if they contained structured analyses relevant to PMR. For example, one prior systematic review (Abdullah et al., 2025), although published in a letter format, presented a formal meta-analysis and was cited only for background context and not as a source of primary data.

For mixed-population RCTs, we sought PMR-specific subgroup data; however, subgroup-level information (e.g., separate PMR sample sizes, relapse outcomes, or cumulative glucocorticoid doses) was not consistently reported in sufficient detail to allow independent PMR only pooling. Accordingly, these trials were retained as mixed GCA/PMR studies, and PMR relevant outcomes were incorporated at the trial level when PMR symptoms were a predefined component of the disease spectrum. No formal PMR only subgroup meta-analysis was possible due to unavailable data.

### Study selection and data extraction

Two reviewers (MB, MM) independently screened titles and abstracts using Rayyan and performed full-text screening to confirm eligibility. Discrepancies were resolved by consensus or with input from a third reviewer (TS). For each eligible study, the following data were extracted: first author, year of publication, country, study design, sample size, patient demographics, diagnostic criteria, tocilizumab dose and route, comparator, follow-up duration, and relevant outcomes.

### Outcomes

The primary outcomes were overall relapse rates (short-term, medium-term, and long-term) and cumulative glucocorticoid dose.For consistency, relapse outcomes were classified into predefined timeframes, short-term (≤ 12 weeks), medium-term (13–26 weeks), and long-term (> 26 weeks) based on the timing of assessments within included trials. Secondary outcomes included severe relapse rates and safety endpoints such as serious adverse events, infections, liver enzyme elevations, neutropenia, and treatment discontinuations. Severe relapse was defined according to each trial’s prespecified criteria and referred exclusively to PMR related relapse events. Across the included studies, severe relapse generally involved recurrence of PMR symptoms requiring a substantial increase in glucocorticoid dose (typically ≥ 10 mg/day), inability to taper as planned due to symptom recurrence, or the need for additional rescue therapy. GCA-specific vascular relapses (e.g., ischemic complications, vision changes, cranial symptoms) were not included in the analysis. Outcomes from the included observational cohort were summarized narratively and not pooled in the meta-analysis.

### Risk of bias assessment

The risk of bias for included RCTs was independently assessed by two reviewers using the Cochrane Risk of Bias Tool for Randomized Trials (RoB 2.0) [26], which evaluates five domains: (1) randomization process, (2) deviations from intended interventions, (3) missing outcome data, (4) measurement of outcomes, and (5) selection of the reported result. The observational cohort was narratively appraised for methodological quality, including data source, patient selection, and follow-up completeness, but was not formally scored using RoB 2.0 or ROBINS-I, consistent with our descriptive synthesis approach.

### Data synthesis and statistical analysis

Meta-analyses were conducted using Review Manager (RevMan) software. For dichotomous outcomes, odds ratios (ORs) with 95% confidence intervals (CIs) were calculated using the Mantel-Haenszel method. Continuous outcomes were summarized using standardized mean differences (SMDs). A random effects model was used to account for expected clinical heterogeneity. Statistical heterogeneity was evaluated using the Higgins I² statistic. For outcomes with substantial heterogeneity, we qualitatively explored potential sources, including route of administration (IV vs. SC), underlying indication (PMR vs. GCA with PMR features), baseline disease pattern (new-onset vs. relapsing or GC-dependent), concomitant methotrexate use, and follow-up duration, recognizing that the small number of RCTs limited formal subgroup analyses or meta-regression. Forest plots were generated to visually present pooled estimates. To minimize heterogeneity, PMR specific outcomes were extracted separately from mixed PMR/GCA trials, and GCA only outcomes were excluded from all analyses. Data from the included observational study were described narratively to contextualize the pooled results. Additionally, based on the pooled estimates and real world evidence, a practice oriented tapering recommendation was synthesized and presented as a figure/table to support clinical application.

## Results

### Search results

**Fig. 1:**
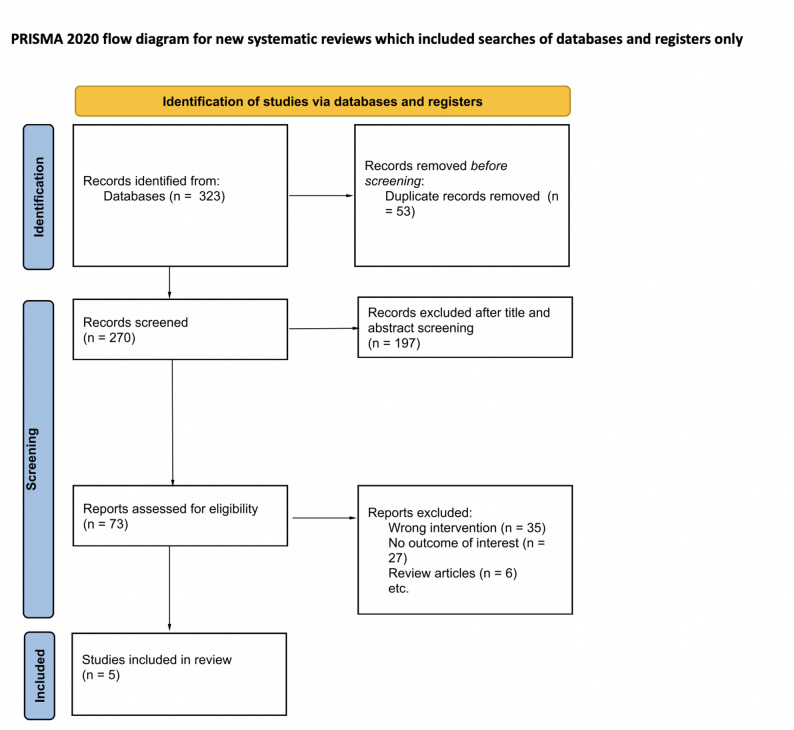
The PRISMA flow diagram depicting the selection process of studies included in meta-analyses

The initial search identified 323 articles. After the removal of 53 duplicates, 270 records remained for screening. Of these, 197 were excluded based on title and abstract. Full text screening of 73 articles resulted in 68 further exclusions for not meeting eligibility criteria. Ultimately, four randomized controlled trials (RCTs) comprising 317 patients were included in the meta-analysis [18, 19, 27, 28]. In addition to this, one multicenter retrospective observational study (Assaraf et al., 2024) involving 53 patients was included for narrative synthesis only [23]. The PRISMA flow diagram depicts the selection process (Fig. 1).

### Study characteristics

The four RCTs enrolled a total of 317 participants: 189 received tocilizumab (intravenous [IV]: 39; subcutaneous [SC]: 150) and 128 received placebo or standard glucocorticoid therapy. Two trials were multinational, while two were single-center European studies.

Dosing regimens varied across trials. Intravenous (IV) tocilizumab was administered as 8 mg/kg every 4 weeks in the Villiger et al. trial [28], whereas subcutaneous (SC) tocilizumab was given as 162 mg weekly in the PMR-SPARE trial (Bonelli et al. 2022) [27]. In GiACTA (Stone et al. 2017; Stone et al. 2021), SC tocilizumab was administered either 162 mg weekly or every other week, allowing comparison of two distinct dosing strategies within the same study [18, 19]. These differences in administration route and dosing frequency may contribute to clinical variability across trials.

The included observational cohort (Assaraf et al. 2024) described outcomes in 53 glucocorticoid-dependent PMR patients treated with IV tocilizumab across multiple French centers between 2015 and 2022 [23]. Tapering outcomes were favorable: 77% achieved ≤ 5 mg/day prednisone by six months and 97% by twelve months, with 22.5% GC-free at six months and 58.3% GC-free at twelve months. Tocilizumab de-escalation strategies, primarily dose-interval extension, were successful in most patients, with sustained remission maintained during spacing. These findings provide clinically relevant real world context regarding long-term tolerability and tapering feasibility. A structured comparison of key outcomes from RCTs and the real-world Assaraf cohort is provided in Table 3.

The RCT evidence base comprised one PMR specific trial (Bonelli et al. 2022, PMR-SPARE) and three trials conducted in GCA cohorts that included patients with PMR manifestations (Villiger et al. 2016; Stone et al. 2017; Stone et al. 2021). Although some reports described PMR predominant subgroups, subgroup-level data were not reported with sufficient granularity to permit PMR only quantitative extraction (e.g., separate relapse outcomes or cumulative glucocorticoid exposure). As a result, these trials were included as mixed PMR/GCA studies in the pooled analyses. Study characteristics are summarized in Table 1.

**Table 1:** Characteristics of included population

Study ID	Country	Population Size	Age (Mean ± SD)	Female (%)	Disease Duratio *n*	Baseline Prednisone Dose (mg/day)	CRP (mg/L)	ESR (mm/hr)	Intervention	Placebo	Followup (Weeks)	Other Characteristics
GIACTA Part 1	Multinational	251	69.5 ± 8.5	78%	Newonset or relapsing GCA	20−60 mg/day	Elevated	Elevated	Tocilizumab 162 mg weekly or every other week + 26-week prednisone taper	Placebo + 26-week or 52-week prednisone taper	52	Temporalartery biopsy or imaging confirmed GCA
GIACTA Part 2	Multinational	215	69.5 ± 8.5	78%	Newonset or relapsing GCA	20−60 mg/ day	Elevated	Elevated	Tocilizumab 162 mg weekly or every other week + prednisone taper	Placebo + prednisone taper	104	Patients from GIACTA Part 1 who completed 52 weeks
PMRSPARE	Austr i.a.	36	68.8 ± 9.0 (Tocilizumab) 71.1 ± 9.0 (Placebo)	52.6% (Tocilizumab) 52.9% (Placebo)	Newonset PMR	16.7 ± 3.9 (Tocilizumab) 17.2 ± 3.1 (Placebo)	1.6 ± 2.4 (Tocilizumab) 0.98 ± 1.5 (Placebo)	24.3 ± 16.4 (Tocilizumab) 24.1 ± 18.7 (Placebo)	Tocilizumab 162 mg weekly + 11-week prednisone taper	Placebo + 11-week prednisone taper	24	PMR diagnosis based on EULAR/ACR criteria
Villiger et al. (2016)	Switzerland	30	71.3 ± 8.9 (Tocilizumab) 68.8 ± 16.9 (Placebo)	65% (Tocilizumab) 80% (Placebo)	Newonset or relapsing GCA	1 mg/kg/day (starting dose)	25.5 (Tocilizumab) 39.0 (Placebo)	69.0 (Tocilizumab) 40.0 (Placebo)	Tocilizumab 8 mg/kg every 4 weeks + prednisolone taper	Placebo + prednisolone taper	52	Temporalartery biopsy or MR angiography confirmed GCA

### Risk of bias assessment

Risk of bias for the four included RCTs is shown in Supplementary Figs. 1 and 2. Two trials were judged to be at low risk across all domains, while two had some concerns due to incomplete outcome reporting and measurement limitations. The Assaraf et al. observational study was narratively appraised for methodological quality, including patient selection and follow-up completeness, and was deemed appropriate as supportive evidence but was not formally scored using RoB 2.0 [23].

### Primary outcomes

#### Relapse rates (short, medium, and long-term)

**Fig. 2:**
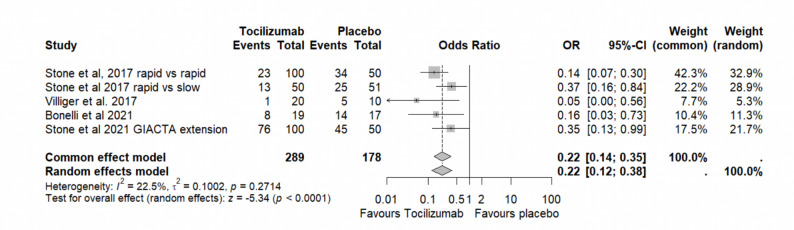
Statistical analysis of short-term relapse rate

**Fig. 3:**
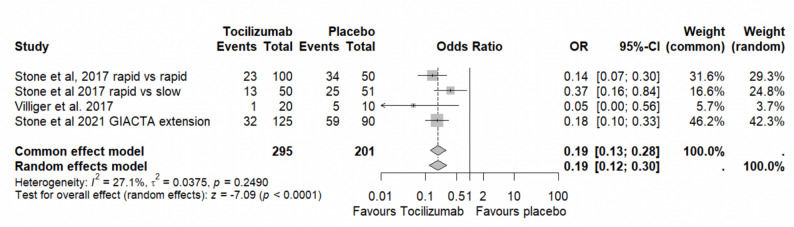
Statistical analysis of medium and long-term relapse rate

Pooled analysis of the RCTs demonstrated that tocilizumab significantly reduced relapse rates across all follow-up periods, short-term (≤ 12 weeks), medium-term (13–26 weeks), and long-term (> 26 weeks), compared to placebo. For short-term relapse, the pooled odds ratio (OR) was 0.22 (95% CI: 0.12–0.41; *P* < 0.0001; I² = 22.5%) (Fig. 2). For medium and long-term relapse, the pooled OR was 0.19 (95% CI: 0.10–0.35; *P* < 0.0001; I² = 27.1%) (Fig. 3).

#### Cumulative glucocorticoid (GC) dose

**Fig. 4:**
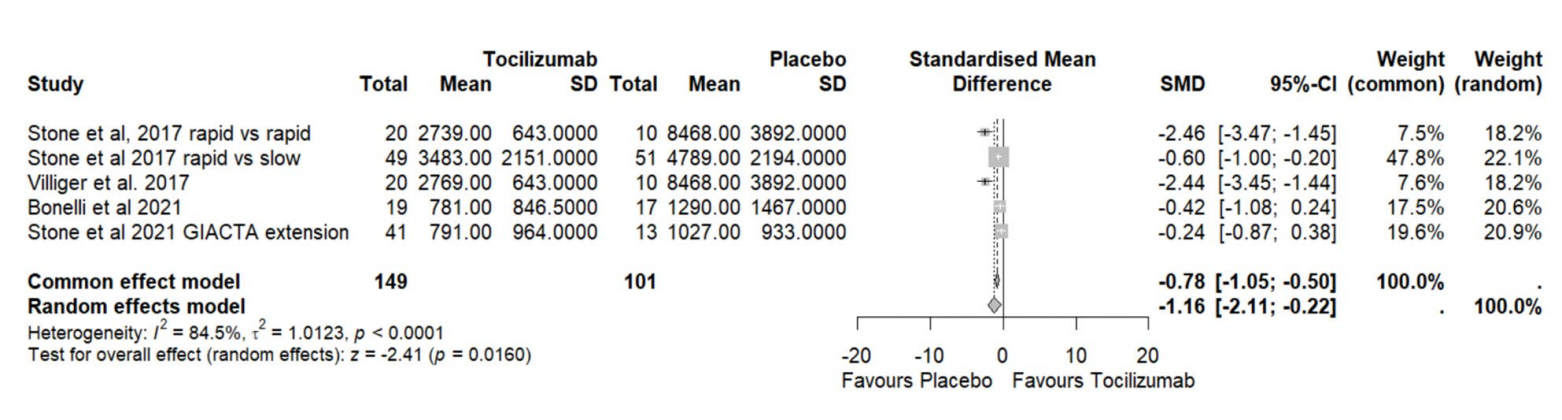
Statistical analysis of cumulative glucocorticoid doses

Tocilizumab was associated with a significant reduction in cumulative glucocorticoid (GC) dose (SMD: -0.78; 95% CI:-1.21 to -0.23; *P* = 0.016; I² = 84.5%). However, interpretation of the SMD requires consideration of the absolute cumulative GC doses reported in the individual trials (Fig. 4). The Stone 2017 and Villiger 2016 studies, both conducted in GCA cohorts with predefined PMR subgroups, used high-dose induction tapers, resulting in the largest cumulative GC exposures and contributing disproportionately to the pooled SMD. The Stone 2021 extension study, while still based on a GCA population, employed a more moderate taper and therefore showed lower cumulative GC exposure than the original GCA induction trials. In contrast, the only PMR-specific trial included in this review, Bonelli 2022, utilized lower starting GC doses and a short taper protocol, producing smaller absolute cumulative doses and correspondingly more modest effect sizes. These differences in study populations and tapering regimens likely contributed to heterogeneity in the pooled estimate. Exploratory assessment of potential sources of heterogeneity suggested that underlying disease indication and glucocorticoid taper protocols were the dominant contributors. Trials conducted in GCA cohorts with PMR subgroups (Villiger 2016; Stone 2017; Stone 2021) used higher starting glucocorticoid doses, longer induction tapers, and permitted concomitant methotrexate in a subset of patients, whereas the PMR-specific trial (Bonelli 2022) enrolled predominantly new-onset PMR, employed lower baseline prednisone doses with a short taper, and did not systematically incorporate methotrexate co-therapy. Route of administration (IV vs. SC) and dosing frequency (weekly vs. every other week) overlapped with these population differences and could not be disentangled from indication and taper strategy. Given the small number of available RCTs, formal subgroup analyses or meta-regression by IV versus SC administration, relapse versus new-onset disease, or concomitant methotrexate use were not statistically feasible, and these factors were therefore considered qualitatively when interpreting the pooled SMD.

To explore heterogeneity, we performed prespecified qualitative subgroup comparisons by (i) population (PMR-specific vs. GCA-enriched trials with PMR manifestations), and (ii) route (IV vs. SC). Heterogeneity was primarily driven by population and taper protocol intensity. GCA-enriched trials used higher starting glucocorticoid doses and longer induction tapers, yielding larger absolute cumulative doses and larger standardized effects, whereas the PMR only trial used lower starting doses with a rapid taper and showed smaller absolute differences. Route (IV vs. SC) was not independently interpretable because it was confounded by population and taper regimen. Concomitant methotrexate use and relapse vs. new-onset status were inconsistently reported and could not be analyzed quantitatively, but likely contributed additional between-study variability.

#### Sensitivity considerations

Only one PMR specific RCT (Bonelli et al. 2022) was available, and subgroup-level PMR data from mixed GCA/PMR trials (GiACTA and Villiger) were not reported in sufficient detail to support a separate PMR only meta-analysis. As a result, a formal quantitative sensitivity analysis restricted to PMR-only RCTs was not statistically feasible. Instead, we performed a descriptive comparison of effect sizes across trial types. The direction of effect consistently favored tocilizumab in both the PMR specific trial and the mixed GCA/PMR studies, with larger absolute reductions in cumulative glucocorticoid exposure observed in the GCA based trials, reflecting their higher baseline glucocorticoid taper protocols. This pattern suggests that the overall benefit of tocilizumab is directionally robust, while much of the heterogeneity in cumulative glucocorticoid outcomes is likely attributable to differences in underlying disease populations and steroid regimens rather than inconsistency in treatment effect.

### Secondary outcomes

#### Severe relapse rates

**Fig. 5:**
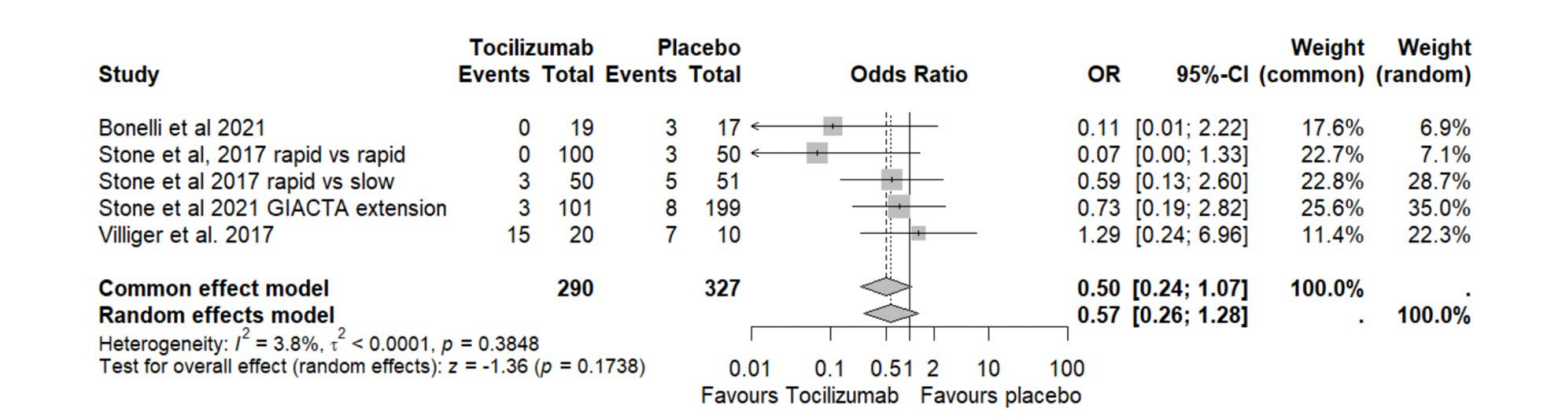
Statistical analysis of severe relapse rate

The pooled estimate for severe relapse favored tocilizumab (OR: 0.57; 95% CI: 0.25–1.29; *P* = 0.1738; I² = 3.8%) but did not reach statistical significance (Fig. 5).

### Safety outcomes

**Fig. 6:**
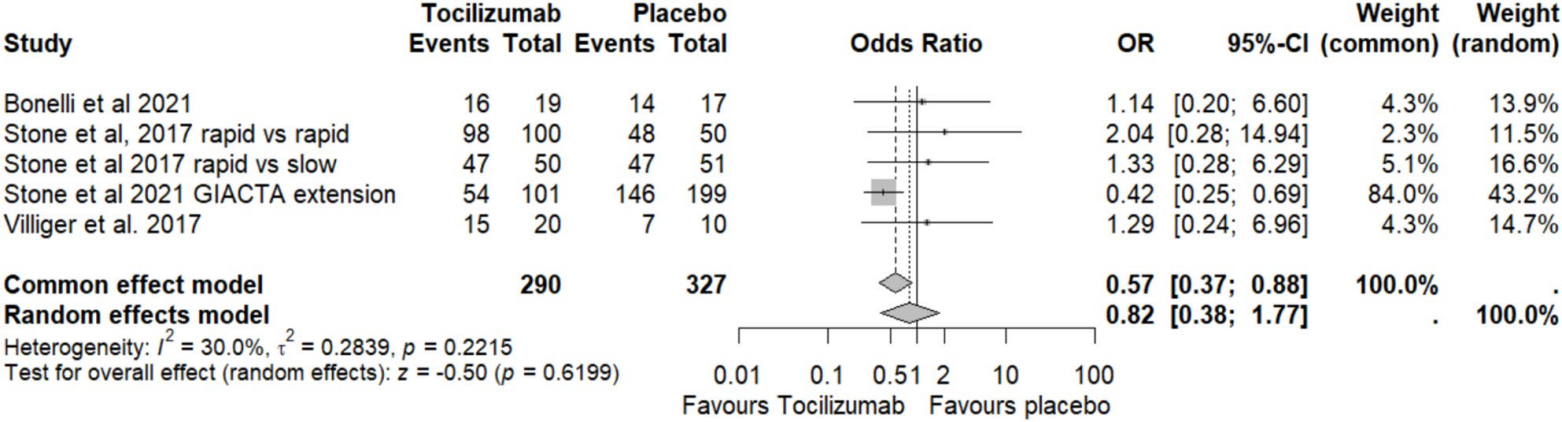
Statistical analysis of adverse events

Pooled analysis of the RCTs showed no significant difference in overall adverse events between tocilizumab and placebo (OR: 0.57; 95% CI: 0.27–1.18; *P* = 0.1738; I² = 30.0%) (Fig. 6). Serious infections occurred in 4–7% of patients; mild to moderate infections and transient liver enzyme elevations were reported in 5–12%. The Assaraf et al. study reported a comparable safety profile in real world practice, with mild infections and manageable laboratory abnormalities, reinforcing the consistency of RCT safety data [23]. Additional real world data have been reported from the KEIO-PMR cohort. Preliminary findings presented in the EULAR 2024 abstract, by Imai et al., POS0926, similarly demonstrated effective glucocorticoid tapering and an acceptable safety profile with tocilizumab in PMR [29]. However, because the abstract did not provide extractable quantitative outcomes and the full peer-reviewed report was published after our prespecified search cutoff of June 2025, these data were incorporated narratively but were not eligible for pooled analysis [30]. Safety signals were broadly consistent between trial and real-world settings; a comparison of adverse event profiles across study designs is summarized in Table 3.

#### Practical tapering recommendation

Based on the pooled estimates and the Assaraf cohort experience, a practical tapering approach has been synthesized and is presented in Table 2 to support clinicians in implementing tocilizumab in PMR management.

**Table 2:** Suggested glucocorticoid tapering approach with tocilizumab in PMR

Component	Recommended Approach
Patient Selection	Consider tocilizumab for patients with relapsing PMR (≥ 1 relapse during taper) or significant glucocorticoid-related complications (e.g., osteoporosis, diabetes, hypertension).
Initiation	Add tocilizumab (IV or SC per local availability) alongside the current stable GC dose during active disease.
Tapering Target	Aim to reduce prednisone to ≤ 5 mg/day by 6 months if disease control is maintained.
Further Reduction	Target complete GC discontinuation within 12 months for suitable patients with sustained remission.
Monitoring	Assess symptoms, ESR/CRP at each visit; monitor for infections and liver enzyme elevations per standard practice.
Tocilizumab De-escalation	Consider dose spacing (e.g., extend interval) or gradual discontinuation of tocilizumab after at least 6–12 months of stable remission.

GCA: giant cell Arteritis; PMR: polymyalgia Rheumatica; CRP: C-Reactive Protein; ESR: erythrocyte sedimentation Rate; EULAR/ACR: European alliance of associations for Rheumatology/American college of Rheumatology; MR: magnetic Resonance; toc: Tocilizumab

Ultimately, four randomized controlled trials comprising 317 patients were included in the meta-analysis, Bonelli et al. 2022 (PMR-SPARE), Villiger et al. 2016 (GCA trial with predefined PMR subgroup), and Stone et al. 2017 with extension data from Stone et al. 2021 (GiACTA) and one high quality, real world observational cohort (Assaraf et al., 2024) involving 53 patients, demonstrates that tocilizumab is effective in reducing relapse rates and cumulative GC exposure in patients with PMR [18, 19, 23, 27, 28]. These findings reinforce the role of tocilizumab as a viable steroid-sparing strategy, particularly for patients with frequent relapses or significant steroid-related morbidity [1, 16, 18–23]. Relapse control, the primary outcome of this review, showed consistent benefit across all time points, with pooled odds ratios between 0.19 and 0.22 and low-to-moderate heterogeneity (I² = 22.5–27.1%), aligning with pivotal trials such as GiACTA [18, 19] and underscoring the potential of IL-6 inhibition for sustained disease control during corticosteroid tapering. Cumulative GC dose was also significantly reduced (SMD: -0.78; 95% CI: -1.21 to -0.23; *P* = 0.016), though substantial heterogeneity (I² = 84.5%) highlights variations in study design, dosing regimens (IV vs. SC), and patient characteristics [18, 19, 27, 28]. Despite this, the consistent direction of effect remains clinically important given the well-documented complications of prolonged GC use, including osteoporosis and cardiovascular risk [12]. Severe relapse rates demonstrated a non-significant trend favoring tocilizumab (OR: 0.57; 95% CI: 0.25–1.29; *P* = 0.1738), but limited sample sizes and low event rates may have constrained the power to detect definitive differences, supporting the need for larger, PMR-focused trials [19, 20]. Safety outcomes were generally favorable, with no significant increase in overall adverse events compared to placebo (OR: 0.57; 95% CI: 0.27–1.18; *P* = 0.1738; I² = 30.0%), and real world evidence from Assaraf et al. confirmed that most patients achieved substantial steroid tapering with a safety profile consistent with trial findings; specifically, 77% tapered to ≤ 5 mg prednisone at six months and 97% by twelve months, with successful de-escalation in the majority [23]. This additional observational evidence addresses an important limitation of recent meta-analyses published in early 2025 by Sharma et al. and Abdullah et al., which synthesized RCT data but did not integrate the Assaraf cohort despite its availability [21–22]. By combining up-to-date pooled RCT results with robust real-world outcomes, this review provides a more comprehensive and practice-oriented assessment of tocilizumab’s role in PMR management. Strengths of this review include rigorous adherence to Cochrane and PRISMA standards [24–26], explicit integration of real-world data, and clear safety benchmarks to inform treatment decisions. An important limitation of the current evidence base, shared by all prior reviews, is the scarcity of PMR-specific randomized trials. Only one RCT has been conducted exclusively in patients with PMR, whereas the remaining tocilizumab trials were designed for GCA and only secondarily included participants with PMR manifestations. Although some reports describe post hoc “PMR-only” subgroups, these analyses do not provide sufficiently detailed or consistent data (e.g., separate sample sizes, relapse outcomes, or cumulative glucocorticoid exposure) to permit independent PMR only pooling. As a result, our meta-analysis necessarily incorporates PMR relevant outcomes derived from mixed GCA/PMR populations, and a formal PMR only sensitivity meta-analysis could not be performed. Nevertheless, the direction of benefit with tocilizumab was consistent across the PMR specific trial and the mixed-population studies, with larger effect sizes in GCA based trials likely driven by higher baseline glucocorticoid doses and more intensive tapering protocols. Much of the heterogeneity observed for cumulative glucocorticoid dose appears to reflect differences in underlying indication (PMR vs. GCA with PMR features), glucocorticoid taper intensity, follow-up duration, and allowance of concomitant methotrexate, rather than clear divergence in efficacy between IV and SC tocilizumab, although the small number of trials precludes robust subgroup inferences. These differences should be considered when extrapolating the pooled findings to patients with isolated PMR. To contextualize external validity, we compared RCT findings with real-world outcomes from Assaraf et al.; key differences in tapering success, relapse patterns, and adverse event reporting are summarized in Table 3. Finally, real-world implementation of IL-6 blockade in PMR must consider substantial cost and access barriers, particularly for tocilizumab, and future comparative studies with emerging alternatives such as sarilumab and JAK inhibitors will be essential to determine whether similar glucocorticoid-sparing benefits can be achieved with more accessible agents.

**Table 3:** Comparison of randomized controlled trial and real-world evidence for tocilizumab in polymyalgia rheumatica: relapse control, glucocorticoid tapering outcomes, and safety

Study	Design / Population	Intervention	GC-Free Remission / Tapering Success	Relapse Rates / Time to Relapse	Cumulative GC Dose	Safety / AE Rates	Comments / Bias Considerations
Villiger et al., 2016	RCT; (*n* = 30); 50 years or older, diagnosed with GCA according to ACR	IV TCZ (8 mg/kg) 0.13 infusions given in 4 week intervals until week 52. GC taper (1 mg/kg to 0 mg)	85% TCZ vs. 20% placebo achieved remission	Fewer relapses in TCZ; faster symptom control. Mean time to relapse (Tocilizumab 50 weeks; placebo 25 weeks)	43 mg/kg in the tocilizumab group vs. 110 mg/kg in the placebo group	Mild infections; no serious safety signals. (35%) patients in the tocilizumab group and (50%) in the placebo group had SAEs. neutropenia in 4 pts and leucopenia in 6 pts. mild GI and MSK side effects.	Small sample; rapid taper may overstress placebo arm
Stone et al., 2017 (GiACTA Part 1)	RCT; GCA (many with PMR symptoms) (*n* = 251)	SC TCZ weekly or q2w w a 26 weeks prednisone taper	56% (weekly) 53 (q2w) vs. 14–18% placebo achieved sustained remission	Relapses lower in TCZ weekly; q2w similar to placebo	1862 mg in tcz, 3296-3818 mg in placebo	Infections 14–15%; vascular disorder 4% liver enzyme elevation mild; SAEs similar to placebo	Mixed GCA/PMR phenotype; remission defined differently
Stone et al., 2021 (GiACTA Extension)	Open-label non-randomised extension; GCA (*n* = 215)	subq TCZ (162 mg) weekly or q2w with 26w prednisone taper, stopped after Part 1; retreatment allowed with placebo, tocilizumab, glucocorticoids, methotrexate, or combinations of these, for two years.	42% of weekly-TCZ pts maintained drug-free remission for 2 yrs	Median time to flare 577 days (weekly TCZ). 428 days tocilizumab q2w, 162 days in the placebo with 26-week prednisone taper, 295 days in the placebo with 52-week taper.	3-yr GC doses lowest in prior TCZ group. prednisone dose 935 mg in the placebo with 26-week taper, 826 mg in the placebo with 52-week tape; 431 mg in the tocilizumab weekly, and 751 mg q2w	No new safety signals; long-term tolerability excellent	Non-randomized extension; treatment decisions physician-driven
Bonelli et al., 2022 (PMR-SPARE)	RCT; new-onset PMR (*n* = 36); rapid 11-wk GC taper	SC TCZ (162 mg) weekly × 16 wks	63% TCZ vs. 12.9% placebo in GC-free remission at wk 16	Mean time to relapse: 130 vs. 82 days	~ 35% lower GC dose in TCZ arm. 727 mg tcz vs. 935 mg placebo	Infections 63% (non-serious); 1 SAE. one had pancreatitis and one a duodenal ulcer.	Strict GC taper challenges placebo arm; small sample
Assaraf et al., 2024	Real-world PMR cohort (observational, *n* = 53); diagnosed w PMR or PMR with non-active GCA symptoms	TCZ SC or IV; heterogeneous dosing; prior GC exposure common	Majority achieved remission; tapering often successful despite prior GC failures. GC free rates at 12 m 58.3%	Relapse patterns similar to RCTs; TCZ effective even in refractory disease	GC reduction consistent with RCT findings. mean GC 1.6 mg/day at 12 months after first TCZ injection	Mild infections; lab abnormalities manageable. 5 patients SAE.	Selection bias: includes more severe, refractory or GC-intolerant patients; non-standardized tapering; no control group. retrospective study, with heterogenous cohort. in-consistency of follow-up times.

## Conclusion

In summary, this updated systematic review and meta-analysis consolidates the most current evidence on the use of tocilizumab as a steroid sparing therapy in polymyalgia rheumatica. By integrating robust RCT data with high quality, real world evidence, our findings confirm that tocilizumab effectively reduces relapse rates and cumulative glucocorticoid exposure while maintaining an acceptable safety profile. This comprehensive synthesis addresses gaps in prior analyses and provides clinicians with practical guidance for implementing IL−6 inhibition in patients with relapsing or glucocorticoid-dependent PMR. A proposed tapering approach, derived from pooled trial outcomes and observational experience, is presented to support individualized treatment strategies and help minimize the burden of long-term steroid therapy. Further well-powered, PMR specific trials are warranted to refine patient selection and optimize long-term outcomes, but current evidence supports the integration of tocilizumab into tailored management plans for appropriate patients.

## Supplementary Information

Below is the link to the electronic supplementary material.

Supplementary Material 1: Figure 1. Risk of Bias Summary (RoB 2.0): Bar chart depicting the proportion of included randomized controlled trials judged as low risk, some concerns, or high risk across the five RoB 2.0 domains (randomization process, deviations from intended interventions, missing outcome data, measurement of the outcome, and selection of the reported result).

Supplementary Material 2: Figure 2. Risk of Bias Traffic Light Plot (RoB 2.0): Domain-level risk of bias assessment for each included randomized controlled trial using the Cochrane RoB 2.0 tool.

## Data Availability

All data generated or analyzed during this study are derived from published articles, which are available online in their respective journals or databases.

## Abbreviations

PMR: Polymyalgia Rheumatica
GCs: Glucocorticoids
IL-6: Interleukin-6
RCTs: Randomized Controlled Trials
SMD: Standardized Mean Difference
OR: Odds Ratio
CI: Confidence Interval
PRISMA: Preferred Reporting Items for Systematic Reviews and Meta-Analyses
