# Supplementary figures and images for: Steroid-sparing strategies in polymyalgia rheumatica: a systematic review and meta-analysis of tocilizumab with practical guidance for tapering
Source: BMC Rheumatol. 2026 Apr 25;10:48. doi: 10.1186/s41927-026-00625-z (PMC13251128; doi:10.1186/s41927-026-00625-z)

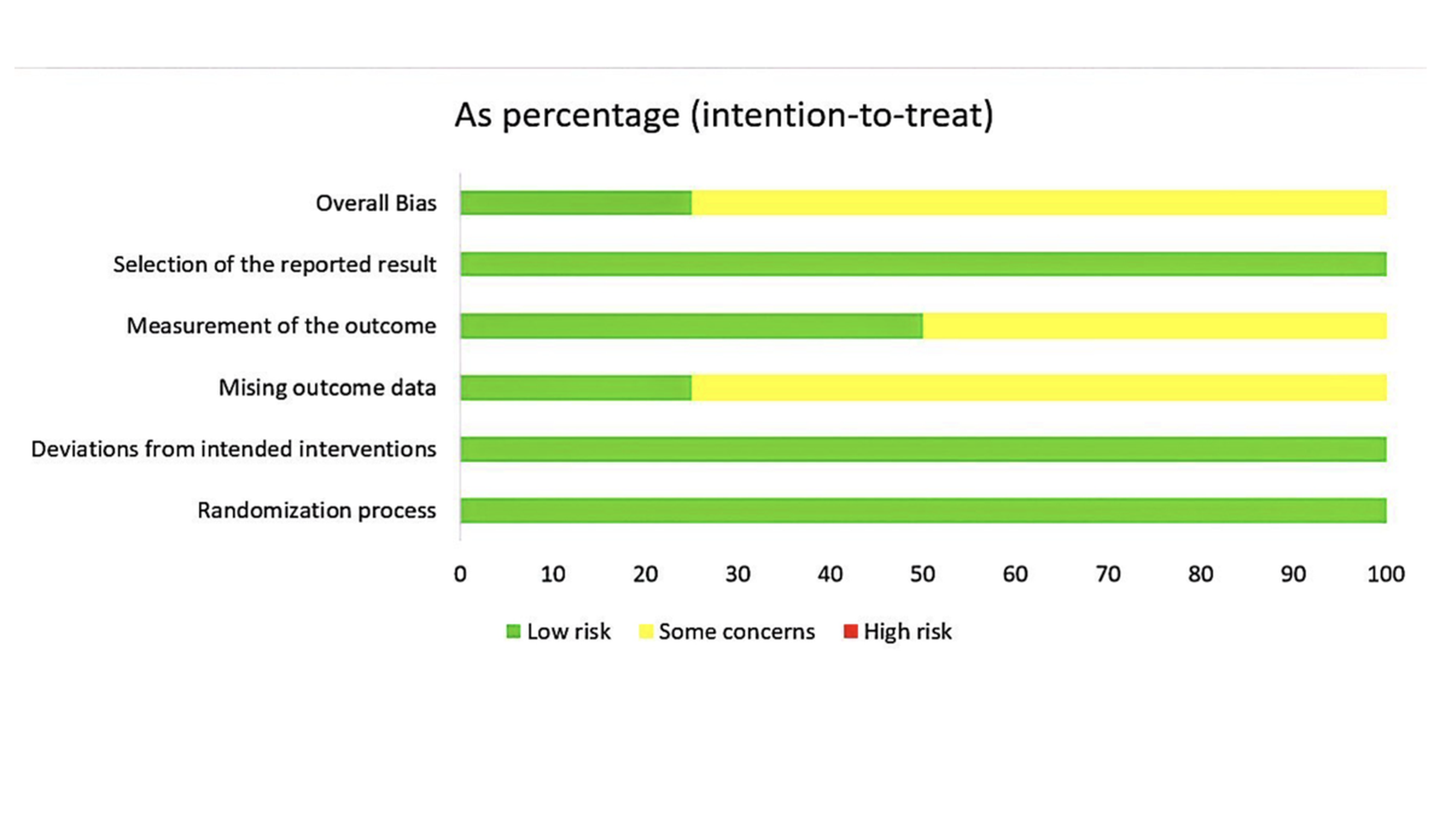

Supplement: Supplementary file 1 — Supplementary Material 1: Figure 1. Risk of Bias Summary (RoB 2.0): Bar chart depicting the proportion of included randomized controlled trials judged as low risk, some concerns, or high risk across the five RoB 2.0 domains (randomization process, deviations from intended interventions, missing outcome data, measurement of the outcome, and selection of the reported result). [file 41927_2026_625_MOESM1_ESM.tiff]

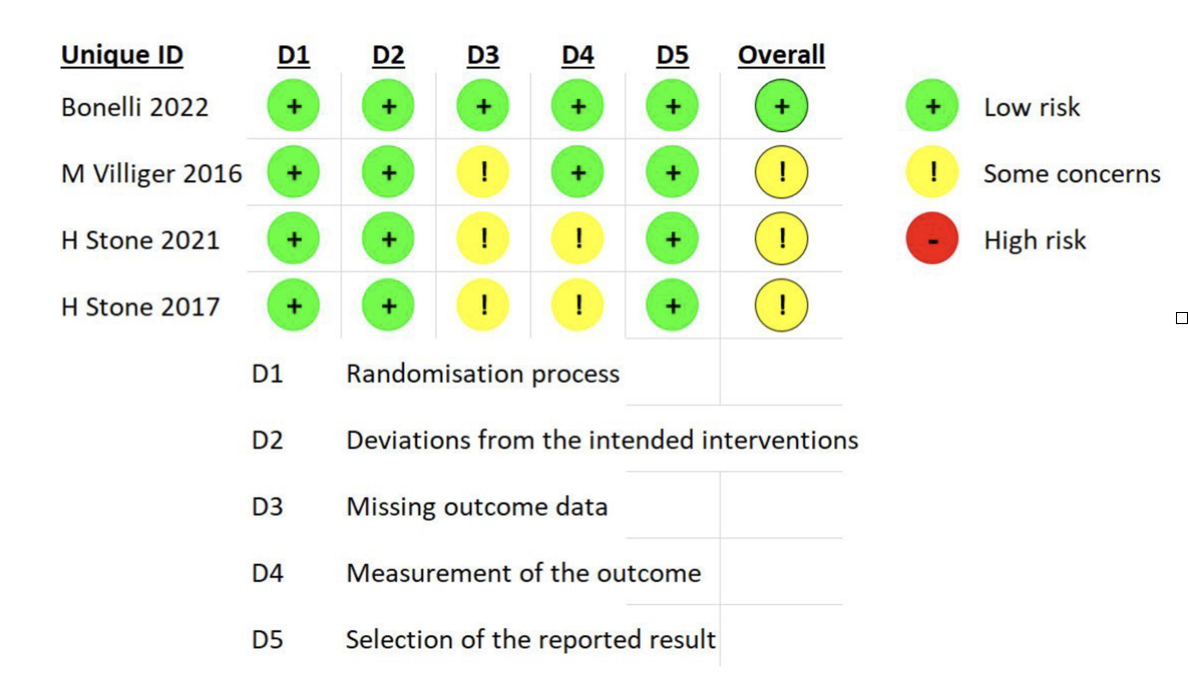

Supplement: Supplementary file 2 — Supplementary Material 2: Figure 2. Risk of Bias Traffic Light Plot (RoB 2.0): Domain-level risk of bias assessment for each included randomized controlled trial using the Cochrane RoB 2.0 tool. [file 41927_2026_625_MOESM2_ESM.tiff]
